# Associations of dietary inflammatory index and composite dietary antioxidant index with erectile dysfunction and the mediating role of metabolic dysregulation: a cross-sectional analysis of NHANES 2001–2004 data

**DOI:** 10.3389/fnut.2025.1538874

**Published:** 2025-06-03

**Authors:** Lang Wang, Can Wei, Junfeng Jing, Dawei Ni, Mingmin Shao, Jingxiong Chen, Wei Wu, Yanbin Zhang

**Affiliations:** ^1^The Second People's Hospital of Hefei, Hefei, China; ^2^The Fifth Clinical School of Medicine, Anhui Medical University, Hefei, China; ^3^College of Humanities and Social Sciences, Shanxi Medical University, Taiyuan, China; ^4^Anhui Provincial Hospital, Hefei, China

**Keywords:** dietary inflammatory index, composite dietary antioxidant index, erectile dysfunction, metabolic dysregulation, triglyceride-glucose index

## Abstract

**Background:**

Inflammation and oxidative stress are a common and fundamental factor in the pathological process leading to erectile dysfunction (ED) and metabolic dysregulation. We aimed to reveal the relationship between the DII, composite dietary antioxidant index (CDAI), and ED, as well as the mediating role of MS, TyG index, MAP, UA, and TC on this relationship.

**Methods:**

This study included 1,488 participants from the NHANES surveys conducted between 2001 and 2004. The DII was constructed using 27 dietary components related to inflammatory potential, while the CDAI was built using six key minerals and vitamins. In the analysis, Spearman correlation, generalized linear models, and weighted logistic regression models were employed. The mediating roles of metabolic indicators in the relationships were investigated using Causal mediation analysis.

**Results:**

After comprehensive adjustment for confounding factors, we found a significant association between DII (OR: 1.07, 95% CI: 1.03–1.11, *p* < 0.001) and CDAI (OR: 0.97, 95% CI: 0.95–1.00, *p* < 0.05) and ED. Additionally, all metabolic indicators except for the TyG index (OR: 1.54, 95% CI: 1.20–1.96, *p* < 0.01), did not show a significant association with the risk of ED. The DII^high^ + CDAI^low^ group had the highest ED risk. Further mediation analysis indicated that TyG played a mediating role between DII and ED, while MS played a mediating role between CDAI and ED.

**Conclusion:**

This study indicates that DII and CDAI were significantly associated with ED.

## 1 Introduction

One of the most prevalent forms of male sexual dysfunction is erectile dysfunction (ED). According to the consensus statement (2015) from the Fourth International Consultation on Sexual Medicine (1), ED is described as the consistent inability to achieve and maintain an erection that is adequate for satisfactory sexual performance, emphasizing that ED is not an occasional, transient phenomenon. A retrospective cross-sectional study reviewing national health and Wellness surveys from 2015 and 2016 across eight countries/regions revealed that the prevalence of ED among adult males in these areas was relatively high, ranging from 37.2% in Brazil to 48.6% in Italy (2). Although ED is not life-threatening, it can have serious negative impacts on mental health anxiety and alexithymia, social relationships (sexual intimacy, career development), and quality of life (3). Recent trends indicate a rising incidence of ED, characterized by an increasingly younger age of onset (4, 5). For younger patients, ED is more often a manifestation of unhealthy lifestyles and psychological stress rather than solely organic disease.

Oxidative stress and inflammation are two closely related biological processes that not only exist independently but also influence each other, jointly affecting the progression of diseases. Inflammatory responses clear damage through proteolytic activity and generate new fluids that support cell growth, promoting tissue regeneration and connective tissue reconstruction; however, oxidative stress exacerbates inflammation and tissue damage by increasing the production of free radicals and depleting antioxidants, both of which can lead to tissue dysfunction (6). In individuals with ED, elevated levels of inflammatory components in the blood, such as TNF-*α*, CRP, IL-1β, IL-6, and IL-18, along with increased oxidative stress markers (ROS and MDA) are common features, and overall antioxidant capacity tends to decline with age (7–11). Chronic inflammation and oxidative stress-induced endothelial dysfunction are among the primary pathological mechanisms of ED (12). Understanding the interplay between inflammation and oxidative stress is crucial for developing new treatment strategies for ED and related conditions.

Diet is not only a fundamental need for human survival but also a crucial component for maintaining health. As mentioned in the Huangdi Neijing (The Yellow Emperor’s Classic of Medicine): “Grains are for nourishment, fruits are for assistance, livestock is for benefit, and vegetables are for supplementation.” A substantial body of epidemiological and clinical studies have shown that diet has extensive impacts on health and disease, including but not limited to ED, diabetes, metabolic dysregulation, neurological disorders, and cancer (13, 14). Thus, dietary interventions are seen as promising adjunct therapies. Given the sensitivity and privacy of ED, many patients prefer non-medical approaches to reduce side effects and enhance sexual performance. Indeed, certain dietary supplements have been utilized to enhance and improve male sexual function (15). Although the results are encouraging, the underlying mechanisms of diet-based therapeutic strategies remain largely unexplored, significantly limiting their widespread application in disease management.

In this study, we utilized the multidimensional and comprehensive NHANES (National Health and Nutrition Examination Survey) database to investigate the combined effects of the DII (dietary inflammatory index) and the CDAI (composite dietary antioxidant index) on ED. To comprehensively reveal their complex interactions in disease development, we also introduced metabolic indicators such as Metabolic score (MS), triglyceride-glucose (TyG) index, mean arterial pressure (MAP), uric acid (UA), and total cholesterol (TC), and examined their mediating roles. Metabolic dysregulation is a common feature of many chronic diseases and can influence levels of inflammation and oxidative stress, and vice versa (16). Our findings may provide new perspectives and strategies for preventing and managing ED.

## 2 Methods

### 2.1 Study participants

**Figure 1:**
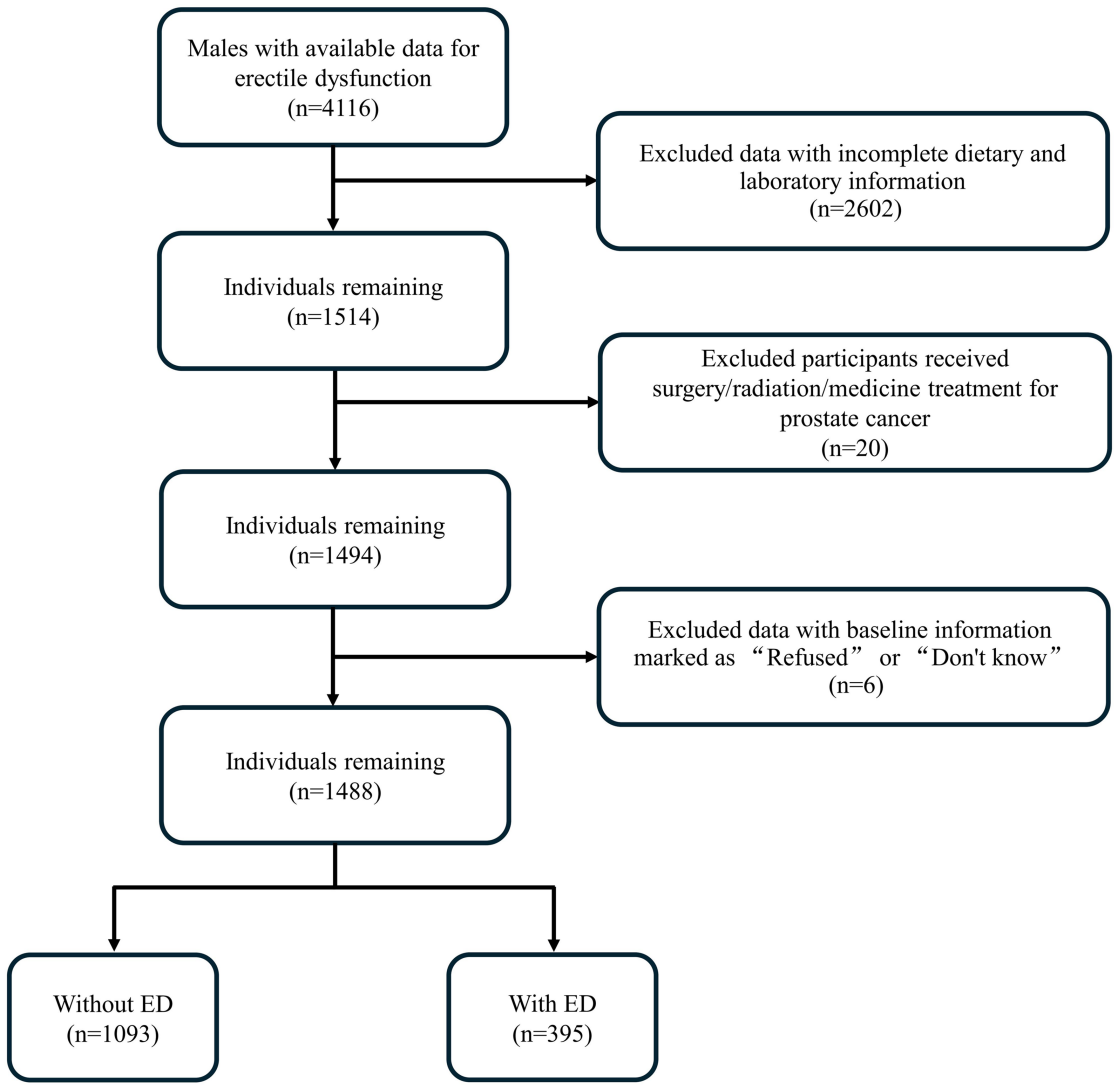
Flow chart for participants.

This cross-sectional study analyzed data from the NHANES from the years 2001 to 2004, the only cycles with available ED data. The initial sample included 4,116 male participants. Participants were excluded if they met either of these conditions: (a) incomplete dietary and laboratory information (*n* = 2,602); (b) history of prostate cancer treatment, including surgery, medication, or chemotherapy (*n* = 20); (c) baseline information marked as “Refused” or “Do not know (*n* = 6). The final analysis included 1,488 participants, with 1,093 classified as non-ED and 395 as ED (Figure 1).

### 2.2 DII

The DII, devised by Shivapp, is a quantitative tool incorporating 45 dietary factors designed to evaluate the association between dietary patterns and health (17). Even when fewer than 30 nutrients are utilized for its calculation, the DII score remains valid. Given the constraints on variety of dietary elements examined in the NHANES, the DII was computed using 27 dietary elements derived from NHANES 2001–2004 data. Previous validation studies have demonstrated that the DII retains predictive validity even when constructed with fewer than 30 components. The calculation of the DII involved these formulas:

DII = (*Z* score’ × the inflammatory effect score of each dietary component).

*Z* score’ = *Z* score (daily mean intake – global daily mean intake)/standard deviation) → (converted to a percentile score) × 2–1.

### 2.3 CDAI

Composite dietary antioxidant index is a comprehensive quantitative tool for assessing the impact of diet on oxidative stress and antioxidant balance. The method for assessing the CDAI, adapted from Wright (18), involves six essential minerals (selenium, zinc) and vitamins (vitamins A, vitamins C, vitamins E, and carotenoids). The global average intake values and corresponding standard deviations for each nutrient were derived from the National Cancer Institute’s dietary reference database, following Wright’s methodology. Standardization was performed by centering individual daily intake values around the global mean and dividing by the global standard deviation to generate standardized z-scores. The CDAI is computed by dividing the difference between the average daily intake and the global average daily intake by the standard deviation.

### 2.4 Definition of ED

The assessment of erectile function was conducted using one specific question: “How would you describe your ability to get and keep an erection adequate for satisfactory intercourse?” Answers included “always or almost always able,” “usually able,” “sometimes able,” or “never able.” For the purposes of this study, ED was characterized as a binary variable. Participants were considered to have ED if they reported being “sometimes able” or “never able” to maintain an erection, while those stating they are “always or almost always able” or “usually able” were considered not having ED.

### 2.5 Metabolic indicators

Measurements of blood pressure were conducted at the MEC. Enzymatic methods were used to measure triglyceride, fasting plasma glucose, UA, and TC levels with an autoanalyzer. To calculate MAP, add 1/3 of the difference between the systolic and diastolic blood pressures to the diastolic pressure. TyG index is determined by taking the natural logarithm of the product of fasting triglycerides and fasting glucose, divided by two, using the average MAP over 2 days. Furthermore, MS was constructed by summing the *z*-converted values of above metabolic indicators.

### 2.6 Covariates

Covariates were selected considering their potential influence on dietary patterns and metabolic outcomes. Participants were classified as low income [poverty ratio (PIR) < 1], medium income (1 ≤ PIR < 4), or high income (PIR ≥ 4). Participants’ smoking status was evaluated using two questions: “Have you smoked at least 100 cigarettes in your entire life?” and “Do you now smoke cigarettes every day, some days, or not at all?” Depending on their answers, individuals were classified as never smokers (smoking < 100 or is a non-smoker), ex-smokers (a history of smoking ≥ 100 cigarettes but was now a non-smoker), or current smokers (currently smokes, either daily or on occasion, with a history of smoking ≥ 100). Physical activity self-reported was divided into moderate or vigorous levels. Covariates such as race/ethnicity, education, and marital status were also included.

### 2.7 Statistical analysis

The CDC guidelines were adhered to for all analyses. The R “mice” package was utilized to impute missing values in the baseline data. The Kruskal–Wallis test was employed to analyze continuous data with skewed distributions, whereas the Chi-square test was used for categorical variables. The associations between DII, CDAI and MS, TyG index, MAP, UA, and TC were assessed by Spearman correlation. Generalized linear models (GLM) evaluated the relationships between DII, CDAI, and metabolic indicators, while binary logistic regression analysis computed the ORs (odds ratios) and corresponding 95% CIs (confidence intervals) related to ED. Three distinct models were applied: Model 1 made no adjustments, Model 2 adjusted for race/ethnicity and age, and Model 3 further adjusted for BMI, PIR, smoking status, physical activity, education, and marital status. Alcohol and total energy intake were not adjusted as they are part of the DII calculation. To examine the joint effects of DII and CDAI on metabolic indicators and ED, both indices were dichotomized at 0, with scores above 0 as high and below 0 as low, forming four subgroups: DII^high^ + CDAI^low^, DII^high^ + CDAI^high^, DII^low^ + CDAI^high^, and DII^low^ + CDAI^low^. The reference group was DII^high^ + CDAI^low^, against which the other subgroups were compared. Causal mediation analysis was performed to examine the mediating role of metabolic indicators, using a GLM and an outcome model (logistic regression). Mediation effects were quantified as the percentage of the indirect effect out of the overall effect, which was tested using Bootstrap sampling (1,000 iterations). To visualize the relationships among them, a Sankey diagram was employed. Sensitivity analyses were performed in two ways: (1) applying a stricter ED definition, which categorized only those who “never achieved or maintained an erection sufficient for satisfactory intercourse” as having ED; and (2) conducting a multiple imputation analysis using chained equations (MICE) to include participants initially excluded for missing dietary and laboratory data. The robustness of the results was assessed by comparing the complete-case and imputed analyses. All statistical analyses incorporated the NHANES sample design, including strata, clusters, and sample weights, to obtain nationally representative estimates. Specifically, the 4-year dietary weights (WTDR4YR) were used for analyses involving dietary variables. Statistical analyses were performed with R software (version 4.2.2) and MSTATA software.^1^ A statistically significant result was shown with a two-sided *p* < 0.05.

## 3 Results

### 3.1 Baseline characteristics of the participants

After exclusions for incomplete ED assessments, missing diet data, and history of prostate cancer treatment, a total of 1,488 participants were included in the study. The majority of participants were non-Hispanic White, with a median age of 44 (IQR: 33, 55). Of these, 395 (26.55%) had ED. Subsequently, we stratified the clinical characteristics of the participants based on the presence or absence of ED, as shown in Table 1. The results indicated that there were statistically significant differences between the ED group and the no-ED group in terms of age, BMI, smoking status, physical activity, education, marital status, dietary quality, and metabolic indicators. Specifically, ED was more prevalent among older adults, those who were obese, had lower educational levels, were unmarried or not in a marital relationship, engaged in low-intensity physical activity, and smoked. Notably, participants without ED had a lower DII (*p* < 0.001) and a higher CDAI (*p* < 0.001). Additionally, participants with ED showed a tendency toward a higher TyG index and metabolic score (MS) (*p* < 0.05), while other metabolic indicators showed no correlation.

**Table 1:** Baseline characteristics of participants.

Characteristics	Total (*N* = 1,488)	No ED (*N* = 1,093)	ED (*N* = 395)	*p*-value
General characteristics				
Age (years)	44.00 (33.00, 55.00)	41.00 (31.00, 51.00)	63.00 (53.00, 72.00)	**<0.001**
BMI (kg/m^2^)	27.47 (24.56, 30.80)	27.32 (24.43, 30.57)	28.17 (25.37, 31.45)	**0.026**
Ethnic, *n* (%)				0.551
Mexican American	332 (7.60%)	239 (7.85%)	93 (6.54%)	
Other Hispanic	50 (3.75%)	35 (3.59%)	15 (4.46%)	
Non-Hispanic White	807 (76.55%)	583 (75.89%)	224 (79.40%)	
Non-Hispanic Black	262 (9.50%)	207 (10.01%)	55 (7.34%)	
Other Race Including Multi Racial	37 (2.59%)	29 (2.66%)	8 (2.26%)	
Income-to-poverty ratio, *n* (%)				0.165
Low income	217 (10.59%)	156 (10.92%)	61 (9.17%)	
Middle income	764 (46.79%)	549 (45.41%)	215 (52.67%)	
High income	507 (42.62%)	388 (43.66%)	119 (38.16%)	
Smoking status, *n* (%)				**<0.001**
Never smoker	601 (42.33%)	493 (45.63%)	108 (28.18%)	
Ex-smoker	476 (29.62%)	280 (25.53%)	196 (47.17%)	
Current smoker	411 (28.05%)	320 (28.84%)	91 (24.65%)	
Physical activity, %				**<0.001**
Moderate	959 (65.80%)	657 (63.10%)	302 (77.41%)	
Vigorous	529 (34.20%)	436 (36.90%)	93 (22.59%)	
Education, %				**0.013**
High school or less	783 (44.62%)	545 (42.30%)	238 (54.53%)	
Some college	406 (31.67%)	321 (33.64%)	85 (23.22%)	
College graduate or higher	299 (23.72%)	227 (24.06%)	72 (22.25%)	
Marital status, *n* (%)				**0.005**
Married/living with partner	446 (32.67%)	353 (34.71%)	93 (23.89%)	
Single/divorced/widowed	1,042 (67.33%)	740 (65.29%)	302 (76.11%)	
Dietary quality index				
DII	−2.67 (−7.38, 0.70)	−3.32 (−8.37, 0.21)	−0.88 (−4.10, 3.03)	**<0.001**
CDAI	0.00 (−4.92, 6.87)	0.71 (−4.18, 8.09)	−2.03 (−6.94, 3.21)	**<0.001**
Metabolic indicators				
MAP, mm Hg	90.00 (83.33, 96.67)	89.67 (83.33, 96.33)	91.00 (83.33, 97.67)	0.482
Uric acid, mg/dL	6.10 (5.40, 7.10)	6.10 (5.40, 7.08)	6.10 (5.30, 7.20)	0.766
Total cholesterol, mg/dL	195.00 (171.00, 222.00)	197.00 (171.00, 224.00)	188.00 (170.11, 215.00)	0.051
TyG index	8.77 (8.35, 9.26)	8.72 (8.32, 9.20)	9.02 (8.58, 9.48)	**<0.001**
MS	0.04 (−1.68, 1.62)	−0.05 (−1.81, 1.61)	0.26 (−1.25, 1.66)	**0.019**

Variables were presented as mean ± SD or median (25th–75th percentile), depending on their distribution, and in numbers (percentages) for categorical variables. Group differences in variables were assessed using the Kruskal–Wallis test or Chi-square test. BMI, Body Mass Index; MAP, mean arterial pressure; UA, uric acid; TC, total cholesterol; TyG, triglyceride-glucose index; MS, metabolic syndrome *Z*-score; DII, dietary inflammatory index; CDAI, composite dietary antioxidant index; ED, erectile dysfunction. Bold values represent statistically significant results (*P* < 0.05).

### 3.2 The association of dietary quality indices (DII, CDAI) with metabolic indicators

Next, we investigated the correlations between DII, CDAI, and metabolic indicators using Spearman correlation analysis. The results, as shown in Supplementary Figure 1, revealed a strong negative correlation between DII and CDAI, with the strongest correlation (*r* = −0.66) in the ED group. For other metabolic indicators, their correlations with DII/(or) CDAI were weak, with most absolute values below 0.1. The relationship between these metabolic indicators and DII/(or) CDAI in the ED group was also more complex. The heatmap also illustrated varying degrees of positive correlation among these metabolic indicators, with high correlations noted between TyG index, MAP, TC, and UA, underscoring the central role of metabolic dysregulation and highlighting the coexistence of multiple metabolic imbalances.

### 3.3 The associations of dietary quality indices (DII, CDAI) and metabolic indicators with ED

**Figure 2:**
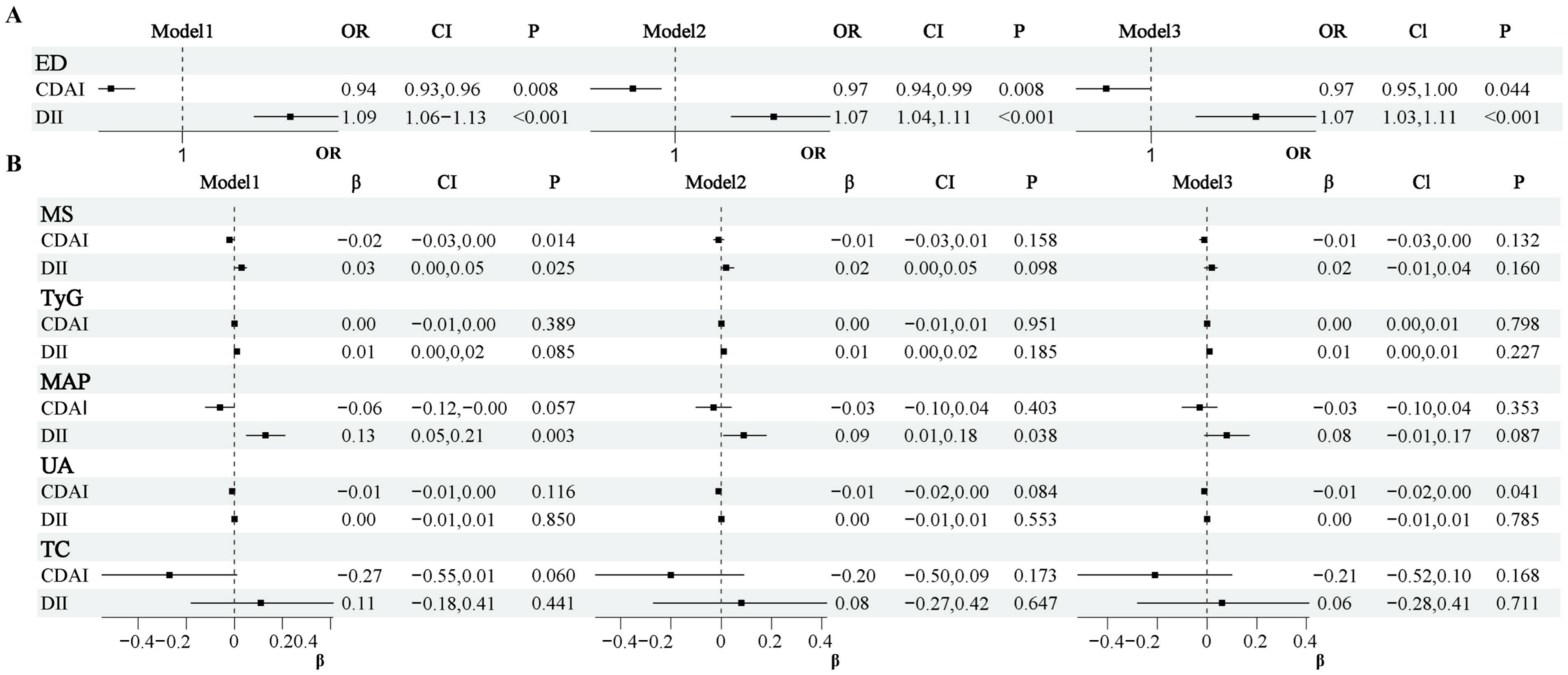
Associations of dietary quality indices (DII, CDAI) with ED **(A)** and metabolic indicators **(B)**. Each point represents the effect estimate (OR or *β*); horizontal lines indicate the 95% confidence interval.

To exclude the influence of confounding factors and provide a more accurate estimation of the true effects of exposure variables DII, CDAI on ED and other metabolic indicators, we sequentially adjusted for different covariates. In all models presented in Figure 2A, both DII and CDAI showed significant associations with ED. After adjusting for all covariates, a higher DII correlated with a 7% higher risk of ED (OR = 1.07, 95% CI: 1.03–1.11, *p* < 0.001), while the risk was reduced by 3% as a result of CDAI (OR = 0.97, 95% CI: 0.953–1.00, *p* < 0.05). The OR values were very similar across the three models, indicating a robust relationship. Additionally, CDAI showed a significant association with UC in model 3 (Figure 2B, *P* < 0.05), although the *β*-coefficient was small.

Additionally, we analyzed the relationships between different metabolic indicators and ED. The results were shown in Table 2. In all three models, the TyG index showed a significant positive correlation with ED (Model 3: *β*-coefficient = 0.43, OR = 1.54, 95% CI: 1.20–1.96, *p* < 0.01). This association suggests that the TyG index may serve as an important metabolic marker in ED. Although the MS showed statistical significance in Model 1 (*β*-coefficient = 0.07, OR = 1.08, 95% CI: 1.02–1.14, *p* < 0.05), it did not show statistical significance in Models 2 and 3. This suggested that the effect of MS may be partially explained by other covariates.

**Table 2:** Associations of metabolic indicators with ED.

Metabolic indicators	*β*	SE	Odds ratio	CI (2.5%)	CI (97.5)	*p*-value
Model 1						
MAP	0.003	0.01	1.00	0.99	1.02	0.623
Uric acid	0.004	0.06	1.06	0.93	1.21	0.358
Cholesterol	−0.002	0.002	1.00	0.99	1.00	0.357
TyG index	0.62	0.11	1.85	1.48	2.32	**<0.001**
MS	0.07	0.03	1.08	1.02	1.14	**0.014**
Model 2						
MAP	−0.01	0.01	0.99	0.97	1.01	0.351
Uric acid	0.13	0.08	1.13	0.96	1.35	0.143
Cholesterol	−0.003	0.003	1.00	0.99	1.00	0.388
TyG index	0.52	0.13	1.69	1.29	2.21	**<0.001**
MS	0.05	0.04	1.05	0.97	1.14	0.186
Model 3						
MAP	−0.01	0.01	0.99	0.97	1.01	0.263
Uric acid	0.10	0.09	1.11	0.93	1.32	0.255
Cholesterol	−0.003	0.003	1.00	0.99	1.00	0.355
TyG index	0.43	0.12	1.54	1.20	1.96	**0.001**
MS	0.03	0.04	1.03	0.95	1.11	0.454

Model 1: unadjusted; Model 2: adjusted for age and race/ethnicity; Model 3: additionally adjusted for BMI, PIR, smoking status, physical activity, education, and marital status. MAP, mean arterial pressure; UA, uric acid; TC, total cholesterol; TyG, triglyceride-glucose index; MS, metabolic syndrome *Z*-score; ED, erectile dysfunction; OR, odds ratio; CI, confidence interval. Bold values represent statistically significant results (*P* < 0.05).

### 3.4 Analysis of combined effects of dietary quality indices (DII, CDAI) on metabolic indicators and ED

After dichotomizing DII and CDAI at a score of 0, four subgroups were formed: DII^high^ + CDAI^low^, DII^high^ + CDAI^high^, DII^low^ + CDAI^high^, and DII^low^ + CDAI^low^. The combined effects of different DII and CDAI combinations on ED were shown in Table 3. Regardless of the combination of DII and CDAI, there was a significant trend toward reduced risk of ED compared to the control group (DII^high^ + CDAI^low^) (Table 3, *p* < 0.05). This suggests that the combination of a high DII and a low CDAI is associated with a higher risk of ED, even after adjusting for multiple confounding factors, where this association remains significant. In Model 1, no associations were observed between different combinations of DII and CDAI and MS, TyG index, MAP, UA, and TC. However, TyG index showed statistical significance after multiple adjustments, with *β*-coefficient of −0.16 in Model 2 (95% CI: −0.28, −0.03, *p* < 0.05) and −0.13 in Model 3 (95% CI: −0.24, −0.01, *p* < 0.05). The TyG index in this joint effect is worth further investigation.

**Table 3:** The combined effects of dietary quality indices (DII, CDAI) on ED and metabolic indicators.

Joint effects	OR (95CIs)	*P*	β (95CIs)	*P*	β (95CIs)	*P*	β (95CIs)	*P*	β (95CIs)	*P*	β (95CIs)	*P*
	ED		MS		TyG index		MAP		UA		TC	
Model 1												
	ref											
AI^high^	0.26 (0.11,0.57)	**0.002**	0.11 (−0.67,0.88)	0.779	0.05 (−0.18,0.29)	0.647	2.15 (−1.60,5.90)	0.252	−0.02 (−0.48,0.44)	0.919	0.73 (−13.59,15.05)	0.918
DII^low^ + CDAI^high^	0.34 (0.23,0.50)	**<0.001**	−0.24 (−0.58, 0.11)	0.174	−0.03 (−0.24,0.18)	0.771	−0.28 (−2.06,1.50)	0.749	−0.06 (−0.29,0.16)	0.585	−1.95 (−9.71,5.82)	0.613
DII^low^ + CDAI^low^	0.45 (0.24,0.81)	**0.010**	−0.27 (−0.72,0.18)	0.225	−0.10 (−0.31, 0.11)	0.334	−0.34 (−2.06,1.39)	0.693	−0.05 (−0.28,0.18)	0.633	0.87 (−8.31,10.06)	0.847
Model 2												
DII^high^ + CDAI^low^	ref											
DII^high^ + CDAI^high^	0.26 (0.12,0.56)	**0.001**	0.21 (−0.52,0.94)	0.562	−0.02 (−0.26,0.22)	0.870	2.40 (−1.15,5.96)	0.177	−0.01 (−0.47,0.45)	0.964	1.63 (−12.53,15.79)	0.816
DII^low^ + CDAI^high^	0.45 (0.30,0.67)	**<0.001**	−0.14 (−0.53,0.25)	0.472	−0.05 (−0.19,0.08)	0.427	0.40 (−1.47,2.27)	0.665	−0.10 (−0.35,0.14)	0.408	−0.95 (−9.30,7.41)	0.819
DII^low^ + CDAI^low^	0.45 (0.24,0.82)	**0.011**	−0.27 (−0.72,0.17)	0.219	−0.16 (−0.28, −0.03)	**0.020**	0.05 (−1.79,1.89)	0.956	−0.10 (−0.33,0.14)	0.411	0.76 (−8.53,10.04)	0.869
Model 3												
DII^high^ + CDAI^low^	ref											
DII^high^ + CDAI^high^	0.28 (0.12,0.65)	**0.004**	0.19 (−0.54,0.92)	0.593	−0.01 (−0.26,0.23)	0.906	2.49 (−0.92,5.89)	0.146	−0.04 (−0.47,0.40)	0.868	1.17 (−12.66,15.00)	0.864
DII^low^ + CDAI^high^	0.49 (0.31,0.76)	**0.003**	−0.10 (−0.49,0.28)	0.583	−0.03 (−0.15,0.09)	0.570	0.56 (−1.37,2.49)	0.560	−0.10 (−0.34,0.15)	0.424	−1.22 (−9.12,6.67)	0.754
DII^low^ + CDAI^low^	0.46 (0.25,0.86)	**0.016**	−0.16 (−0.55,0.23)	0.416	−0.13 (−0.24, −0.01)	**0.031**	0.43 (−1.33,2.19)	0.622	−0.05 (−0.28,0.18)	0.661	1.11 (−7.71,9.93)	0.798

DII and CDAI were dichotomized at 0 to define four joint exposure groups. “DII^high^ + CDAI^low^” was used as the reference group. Model 1: unadjusted; Model 2: adjusted for age and race/ethnicity; Model 3: additionally adjusted for BMI, PIR, smoking status, physical activity, education, and marital status. DII, dietary inflammatory index; CDAI, composite dietary antioxidant index; ED, erectile dysfunction; MS, metabolic syndrome *Z*-score; TyG, triglyceride-glucose index; MAP, mean arterial pressure; UA, uric acid; TC, total cholesterol; OR, odds ratio; CI, confidence interval; β, beta coefficient. Bold values represent statistically significant results (*P* < 0.05).

### 3.5 The influence of metabolic indicators as mediators in establishing the association among DII, CDAI, and ED

**Figure 3:**
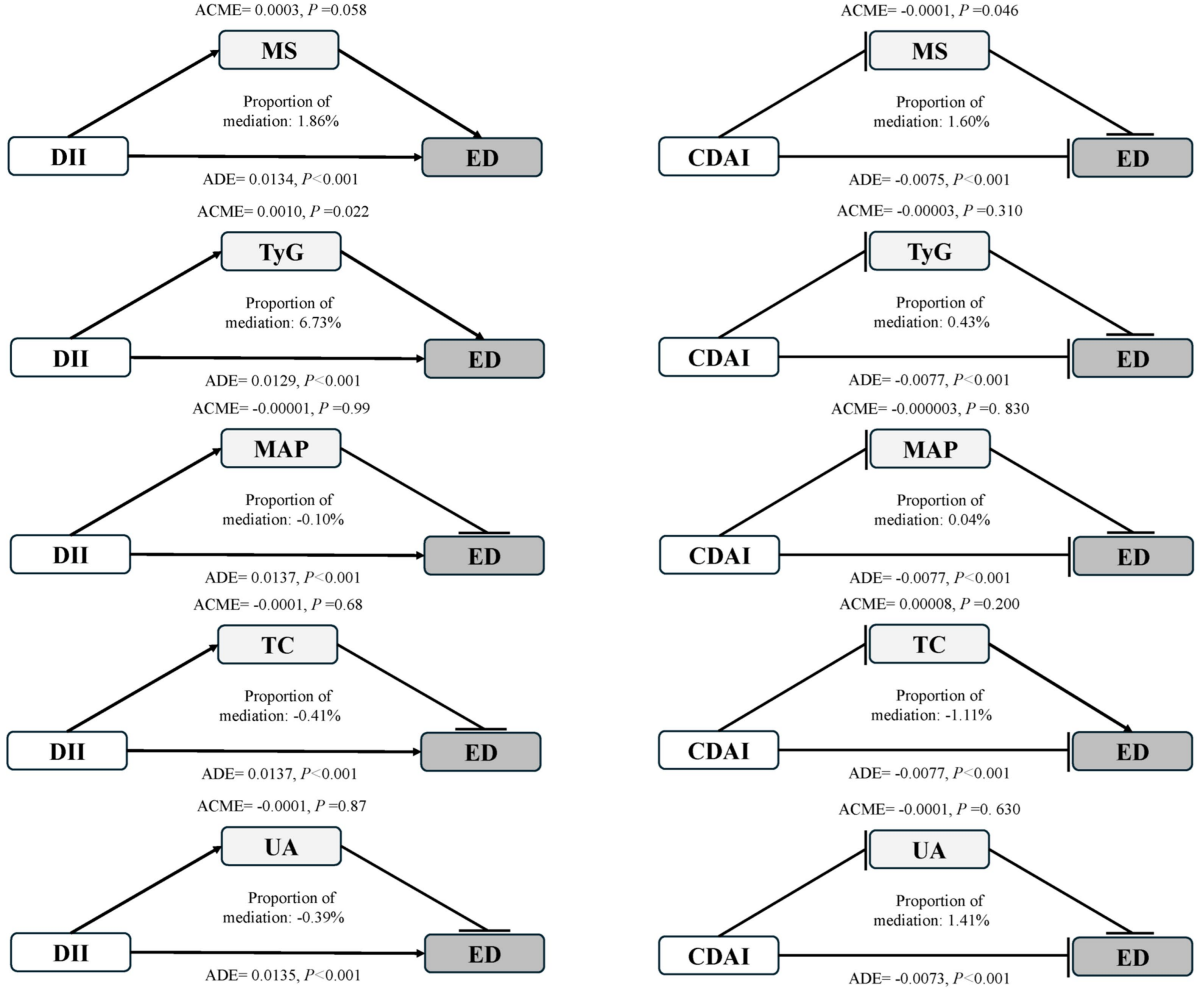
The influence of metabolic indicators as mediators in establishing the association among DII, CDAI, and ED. The left panel shows mediation models with DII as the exposure; the right panel shows CDAI as the exposure. Arrows symbolize promotion, and circle heads signify inhibition. ACME, average causal mediation effects; ADE, average direct effects.

Metabolic indicators MS, TyG, MAP, UA and TC were considered as mediators, and the effects of independent variables DII and CDAI on ED were explored in these mediated models. The results (Figure 3) showed that, TyG mediated 6.73% of the relationship between DII and ED (*p* < 0.05), with and βADE of 0.0129. In the relationship between CDAI and ED, the mediating effect of MS was 1.6%, with βADE of −0.0075. Other metabolic markers did not show significant mediating effects in their respective pathways. Metabolic indicators partially mediate the relationship between DII and CDAI and ED. Furthermore, a Sankey diagram visually illustrated the interaction and potential causality between TyG, DII, MS, and CDAI (Supplementary Figure 2).

### 3.6 Sensitivity analysis

As part of the sensitivity analyses, we conducted multiple imputation for missing baseline data. The results after imputation were consistent with those of the complete-case analysis, showing similar associations between DII, CDAI, metabolic indicators, and ED. Specifically, the odds ratios and effect estimates changed minimally (<5%), indicating that the exclusion of participants with incomplete data did not substantially bias our conclusions. The sensitivity analysis results showed that the relationship between DII and ED, as well as between CDAI and ED remained stable (Supplementary Table 1, *p* < 0.01). Similar results were observed for the association between CDAI and UC (Supplementary Table 1, *p* < 0.05). Furthermore, a significant positive association was also found between TyG index and ED, with a *β*-coefficient of 0.43, OR of 1.54 (95% CI: 1.19–1.98, *p* < 0.01) (Supplementary Table 2).

## 4 Discussion

Diet is a key modifiable target for reducing chronic diseases risk. In clinical trials, dietary therapy has effectively improved or restored ED in patients with obesity or metabolic dysregulation (19, 20). The gluten-free diet has been reported to help improve celiac disease-related sexual dysfunction (21). In our current cross-sectional study, we provided insights into the relationship between the DII, the CDAI, and ED by comprehensively handling data and adjusting for multiple levels of confounding factors. Additionally, metabolic dysregulation (MS and TyG) partially mediated this process. To our knowledge, this study is one of the earliest to specifically combine metabolic indicators with DII, CDAI, and ED risk.

The etiology of ED is highly complex, involving well-defined organic lesions, such as vascular and neurological dysfunction as well as lifestyle and psychosocial factors (22). Consistent with previous studies, unhealthy lifestyles and unstable environmental factors are significantly associated with higher rates of ED (23, 24). Our study also revealed an intriguing finding that ex-smokers exhibited a higher risk of ED when compared with current smokers, despite the lack of statistical significance. This may suggest that those who have quit smoking already have some serious health issues that prompted them to stop smoking.

Although the link between dietary quality and ED has not been fully elucidated, several studies suggest that inflammation and oxidative stress may play important roles. High-fat diet-induced diabetic ED in rats is associated with higher levels of pyroptosis in the corpus cavernosum tissue (25), which may reduce the bioavailability of nitric oxide and cause endothelial damage (26). This, in turn, leads to increased contractile responsiveness of the corpus cavernosum. Given this, recent studies have emphasized the importance of adopting anti-inflammatory and antioxidant diets for the prevention and management of ED. In diabetes-induced testicular morphological damage, omega-3 fatty acids and carotenoids (astaxanthin) found in seafood can exert protective effects through their antioxidant, anti-inflammatory, and anti-apoptotic properties (27). Additionally, a prospective study involving American male health professionals discovered that a higher Mediterranean diet score was linked to a reduced risk of ED (28, 29). As one of the most evidence-based diets, the Mediterranean diet can increase NO bioavailability, enhance antioxidant defense, and suppress inflammation, all of which are crucial for reducing cardiovascular disease risk and, consequently, ED (26). Other studies indicated that both Mediterranean and plant-based diets are negatively correlated with the DII (30). Researches also showed that high dietary lycopene intake can reduce ED risk (31, 32). Furthermore, various dietary antioxidants, such as vitamins C and E and phytochemicals, have positive effects on ED (33–35). These findings strongly support our conclusions. Even after accounting for several confounding factors, the significant association between DII, CDAI, and the probability of ED was remained prominent. Additionally, recent NHANES-based studies have further validated the relevance of dietary quality to ED risk. Liu et al. (36) demonstrated that higher intake of trace metals such as magnesium, zinc, copper, and selenium was associated with lower ED prevalence. Using dose–response curve analysis, multivariate adjustment, and propensity score matching, this study confirmed the protective role of these micronutrients. Similarly, Hao et al. (37) found that a higher Composite Dietary Antioxidant Index (CDAI) was significantly associated with reduced ED risk, even after adjusting for key confounders through propensity score matching. These findings are consistent with our results and reinforce the role of anti-inflammatory and antioxidant dietary components in sexual health. Compared with these prior studies, our study provides a more integrated perspective by jointly assessing both inflammatory (DII) and antioxidant (CDAI) dietary profiles and examining their interaction with metabolic dysfunction as potential mediators of ED. Compared to the Mediterranean diet score, which reflects an overall healthy eating pattern rich in anti-inflammatory and antioxidant foods, the DII and CDAI provide more focused quantitative measures of the inflammatory and antioxidant potential of the diet, respectively. Although these indices differ in scope and calculation, they collectively underscore the critical role of dietary quality in modulating ED risk. Nevertheless, promoting dietary interventions based on these indices faces practical challenges. Adherence to anti-inflammatory or antioxidant-rich diets can be influenced by cultural dietary preferences, socioeconomic status, food accessibility, and individual motivation, all of which vary widely across populations. Mechanistically, individuals with a high DII and low CDAI dietary pattern are likely exposed to a persistent pro-inflammatory and oxidative stress environment. This chronic state may impair endothelial function, reduce nitric oxide bioavailability, promote vascular dysfunction, and ultimately contribute to the pathogenesis of ED. Our findings therefore highlight the potential for targeted dietary modifications to serve as an adjunct strategy in ED prevention and management.

Recent studies have reported cross-sectional associations between DII and CDAI and ED individually (38–40). Although CDAI and DII focus on different dietary factors, their combined study can provide a more comprehensive understanding of diet’s impact on disease. Zheng et al. (41) reported on a study involving Chronic Obstructive Pulmonary Disease. However, up to this point, the synergies between DII and CDAI have largely remained unknown.

Diets rich in anti-inflammatory and antioxidant components may play a role in the prevention and management of ED, suggesting the potential value of dietary interventions as a therapeutic strategy. In clinical practice, DII/CDAI can be used as tools to assess patients’ dietary patterns, allowing for personalized dietary counseling to promote healthier eating changes. However, due to the cross-sectional nature of our study, future longitudinal or interventional studies are needed to explore these potential causal relationships.

Metabolic dysregulation is a cluster of conditions including insulin resistance, central obesity, arterial hypertension, and hyperlipidemia, which can adversely affect male urogenital organs. When diabetes and hypertension coexist, they exacerbate ED by increasing oxidative stress, inflammation, apoptosis, and autophagy, while simultaneously inhibiting eNOS activity and NO production (42). Considering the relationship between metabolic dysregulation and ED, we further investigated the potential mediating role of metabolic dysfunction. Our results indicated an association between ED risk and MS as well as the TyG index, with these factors partially mediating the observed associations among DII, CDAI, and ED. The TyG index, developed by Simental-Mendía et al. (43) is a simple, cost-effective, and easily obtainable tool for assessing insulin resistance (44). Studies have shown that approximately 20% of newly diagnosed ED cases exhibit impaired glucose metabolism (45). Furthermore, other studies have reported a relationship between the TyG index and ED, noting its elevation in ED patients (46). Our findings indicate that, after adjusting for all confounding factors, a one-unit increase in the TyG index is associated with a 54% increase in the prevalence of ED. In states of insulin resistance, decreased efficiency of insulin action can be associated with elevated blood glucose and lipid levels, which are closely related to increased inflammation and oxidative stress (47). These processes may impact the pathophysiological mechanisms of ED. However, while the TyG index mediates the relationship between CDAI and ED, the ADE is small, suggesting that further comprehensive evaluation is needed in practical applications. Although the mediation effects observed for TyG (6.73%) and MS (1.6%) were modest in magnitude, they may still be biologically meaningful. In chronic disease processes such as erectile dysfunction, even small indirect effects can have cumulative impacts at the population level over time. Moreover, our mediation analysis was limited to a few metabolic indicators. Additional biological mediators, including inflammatory biomarkers such as C-reactive protein (CRP) and oxidative stress markers, could play critical roles in the complex pathways linking dietary quality and ED risk. Future studies incorporating a broader set of biomarkers and longitudinal designs are warranted to further elucidate these mechanistic relationships.

In the current study, we utilized representative data from NHANES, ensuring data quality and validity through the exclusion of individuals who had undergone prostate cancer treatment and those with missing baseline information, as well as eliminate confounding factors. Despite focusing on different dietary aspects, the combined study of CDAI and DII provides a more comprehensive understanding of the impact of diet on disease. However, the cross-sectional design of this study fundamentally limits the ability to establish temporal or causal relationships between dietary indices and erectile dysfunction. Although associations were identified, causality cannot be inferred. Therefore, future prospective longitudinal studies and randomized controlled trials are warranted to confirm these findings and elucidate underlying mechanisms. Additionally, the limitations of observational studies mean that other potential influences, such as psychological disorders, cannot be ruled out. Moreover, the data were obtained through recall interviews, which may be subject to patient recall bias and other potential biases. Although a significant proportion of participants were excluded due to missing information, our multiple imputation-based sensitivity analysis demonstrated that the primary findings remained robust. Therefore, selection bias is unlikely to have materially affected our results. Nevertheless, the possibility of residual confounding due to missing variables cannot be entirely ruled out, and future studies with more comprehensive data collection are warranted. Furthermore, certain potential confounders, such as mental health status (e.g., depression, anxiety) and the use of medications like antihypertensives, were not included in our adjustment models due to data limitations. Both psychological factors and specific medications have been associated with erectile dysfunction risk in prior studies. The lack of adjustment for these variables may have introduced residual confounding. Future research with more detailed psychiatric assessments and medication usage data is necessary to further validate these findings.

## 5 Conclusion

Higher DII scores and lower CDAI values were linked to a greater risk of ED, partially mediated by MS and the TyG index.

## Data Availability

Publicly available datasets were analyzed in this study. This data can be found here: National Health and Nutrition Examination Survey (NHANES) for the years 2001–2004. The dataset can be accessed at https://wwwn.cdc.gov/nchs/nhanes/default.aspx. All analyses were performed on these publicly accessible data in compliance with ethical standards.

## Footnotes

^1^ https://www.mstata.com/

## References

[1] McCabe MP Sharlip ID Atalla E Balon R Fisher AD Laumann E. Definitions of sexual dysfunctions in women and men: a consensus statement from the fourth international consultation on sexual medicine 2015. J Sex Med. (2016) 13:135–43. doi: 10.1016/j.jsxm.2015.12.019, PMID: 26953828

[2] Goldstein I Goren A Li VW Tang WY Hassan TA. Epidemiology update of erectile dysfunction in eight countries with high burden. Sex Med Rev. (2020) 8:48–58. doi: 10.1016/j.sxmr.2019.06.008, PMID: 31416758

[3] Elterman DS Bhattacharyya SK Mafilios M Woodward E Nitschelm K Burnett AL. The quality of life and economic burden of erectile dysfunction. Res Rep Urol. (2021) 13:79–86. doi: 10.2147/RRU.S283097, PMID: 33634039 PMC7901407

[4] Nguyen HMT Gabrielson AT Hellstrom WJG. Erectile dysfunction in young men-a review of the prevalence and risk factors. Sex Med Rev. (2017) 5:508–20. doi: 10.1016/j.sxmr.2017.05.004, PMID: 28642047

[5] Mark KP Arenella K Girard A Herbenick D Fu J Coleman E. Erectile dysfunction prevalence in the United States: report from the 2021 National Survey of sexual wellbeing. J Sex Med. (2024) 21:296–303. doi: 10.1093/jsxmed/qdae008, PMID: 38410029

[6] Arulselvan P Fard MT Tan WS Gothai S Fakurazi S Norhaizan ME. Role of antioxidants and natural products in inflammation. Oxidative Med Cell Longev. (2016) 2016:5276130. doi: 10.1155/2016/5276130, PMID: 27803762 PMC5075620

[7] Matos G Hirotsu C Alvarenga TA Cintra F Bittencourt L Tufik S. The association between TNF-α and erectile dysfunction complaints. Andrology. (2013) 1:872–8. doi: 10.1111/j.2047-2927.2013.00136.x, PMID: 24123864

[8] Sivritepe R Uçak Basat S Baygul A Küçük EV. The effect of interleukin-6 level at the time of hospitalisation on erectile functions in hospitalised patients with COVID-19. Andrologia. (2022) 54:e14285. doi: 10.1111/and.14285, PMID: 34687052 PMC8646451

[9] Urios A Ordoño F García-García R Mangas-Losada A Leone P José Gallego J. Tadalafil treatment improves inflammation, cognitive function, and mismatch negativity of patients with low urinary tract symptoms and erectile dysfunction. Sci Rep. (2019) 9:17119. doi: 10.1038/s41598-019-53136-y, PMID: 31745217 PMC6863868

[10] Li C-H Zhao X Xu Y Zhang F Li C-T Zhao S-N. Increased serum asprosin is correlated with diabetes mellitus-induced erectile dysfunction. Diabetol Metab Syndr. (2024) 16:91. doi: 10.1186/s13098-024-01333-9, PMID: 38659035 PMC11044402

[11] Mao Y Zha Y Zang Y Gao Y Sun J Liu Y. Isorhamnetin improves diabetes-induced erectile dysfunction in rats through activation of the PI3K/AKT/eNOS signaling pathway. Biomed Pharmacother. (2024) 177:116987. doi: 10.1016/j.biopha.2024.116987, PMID: 38897159

[12] Kaltsas A Zikopoulos A Dimitriadis F Sheshi D Politis M Moustakli E. Oxidative stress and erectile dysfunction: pathophysiology, impacts, and potential treatments. Curr Issues Mol Biol. (2024) 46:8807–34. doi: 10.3390/cimb46080521, PMID: 39194738 PMC11353036

[13] Cena H Calder PC. Defining a healthy diet: evidence for the role of contemporary dietary patterns in health and disease. Nutrients. (2020) 12:334. doi: 10.3390/nu12020334, PMID: 32012681 PMC7071223

[14] Mishra A Giuliani G Longo VD. Nutrition and dietary restrictions in cancer prevention. Biochim Biophys Acta Rev Cancer. (2024) 1879:189063. doi: 10.1016/j.bbcan.2023.189063, PMID: 38147966

[15] Petre GC Francini-Pesenti F Vitagliano A Grande G Ferlin A Garolla A. Dietary supplements for erectile dysfunction: analysis of marketed products, systematic review, meta-analysis and rational use. Nutrients. (2023) 15:3677. doi: 10.3390/nu15173677, PMID: 37686709 PMC10490034

[16] Masenga SK Kabwe LS Chakulya M Kirabo A. Mechanisms of oxidative stress in metabolic syndrome. Int J Mol Sci. (2023) 24:7898. doi: 10.3390/ijms24097898, PMID: 37175603 PMC10178199

[17] Shivappa N Steck SE Hurley TG Hussey JR Hébert JR. Designing and developing a literature-derived, population-based dietary inflammatory index. Public Health Nutr. (2014) 17:1689–96. doi: 10.1017/S1368980013002115, PMID: 23941862 PMC3925198

[18] Wright ME Mayne ST Stolzenberg-Solomon RZ Li Z Pietinen P Taylor PR. Development of a comprehensive dietary antioxidant index and application to lung cancer risk in a cohort of male smokers. Am J Epidemiol. (2004) 160:68–76. doi: 10.1093/aje/kwh173, PMID: 15229119

[19] da Silva SC da Costa CM Souto JCS Chiogna LM de Albuquerque Santos ZE Rhoden EL. The effects of a low carbohydrate diet on erectile function and serum testosterone levels in hypogonadal men with metabolic syndrome: a randomized clinical trial. BMC Endocr Disord. (2023) 23:30. doi: 10.1186/s12902-023-01278-6, PMID: 36732722 PMC9892661

[20] Defeudis G Mazzilli R Di Tommaso AM Zamponi V Carlomagno F Tuccinardi D. Effects of diet and antihyperglycemic drugs on erectile dysfunction: a systematic review. Andrology. (2023) 11:282–94. doi: 10.1111/andr.13192, PMID: 35485604 PMC10084359

[21] Romano L Pellegrino R Sciorio C Barone B Gravina AG Santonastaso A. Erectile and sexual dysfunction in male and female patients with celiac disease: a cross-sectional observational study. Andrology. (2022) 10:910–8. doi: 10.1111/andr.13186, PMID: 35419983 PMC9324123

[22] MacDonald SM Burnett AL. Physiology of erection and pathophysiology of erectile dysfunction. Urol Clin North Am. (2021) 48:513–25. doi: 10.1016/j.ucl.2021.06.009, PMID: 34602172

[23] Cao S Hu X Shao Y Wang Y Tang Y Ren S. Relationship between weight-adjusted-waist index and erectile dysfunction in the united state: results from NHANES 2001-2004. Front Endocrinol. (2023) 14:1128076. doi: 10.3389/fendo.2023.1128076, PMID: 37181040 PMC10167952

[24] Li S Song J-M Zhang K Zhang C-L. A meta-analysis of erectile dysfunction and alcohol consumption. Urol Int. (2021) 105:969–85. doi: 10.1159/000508171, PMID: 34521090

[25] Luo C Peng Y Zhou X Fan J Chen W Zhang H. NLRP3 downregulation enhances engraftment and functionality of adipose-derived stem cells to alleviate erectile dysfunction in diabetic rats. Front Endocrinol. (2022) 13:913296. doi: 10.3389/fendo.2022.913296, PMID: 35937790 PMC9354456

[26] de Souza ILL Barros BC de Oliveira GA Queiroga FR Toscano LT Silva AS. Hypercaloric diet establishes erectile dysfunction in rat: mechanisms underlying the endothelial damage. Front Physiol. (2017) 8:760. doi: 10.3389/fphys.2017.00760, PMID: 29085300 PMC5649200

[27] Caiaffo V Ribeiro de Oliveira BD de Sa FB Neto JE da Silva Junior VA. Marine food protection in testicular damages caused by diabetes mellitus. Curr Diabetes Rev. (2017) 13:566–72. doi: 10.2174/1573399812666160618123229, PMID: 27324876

[28] Bauer SR Breyer BN Stampfer MJ Rimm EB Giovannucci EL Kenfield SA. Association of Diet with Erectile Dysfunction among men in the health professionals follow-up study. JAMA Netw Open. (2020) 3:e2021701. doi: 10.1001/jamanetworkopen.2020.21701, PMID: 33185675 PMC7666422

[29] Gualtieri P Marchetti M Frank G Smeriglio A Trombetta D Colica C. Antioxidant-enriched diet on oxidative stress and inflammation gene expression: a randomized controlled trial. Genes. (2023) 14:206. doi: 10.3390/genes14010206, PMID: 36672947 PMC9859217

[30] Carto C Pagalavan M Nackeeran S Blachman-Braun R Kresch E Kuchakulla M. Consumption of a healthy plant-based diet is associated with a decreased risk of erectile dysfunction: a cross-sectional study of the National Health and nutrition examination survey. Urology. (2022) 161:76–82. doi: 10.1016/j.urology.2021.12.021 34979217

[31] Gao Y Liu C Lu X Lu K Zhang L Mao W. Lycopene intake and the risk of erectile dysfunction in US adults: NHANES 2001-2004. Andrology. (2024) 12:45–55. doi: 10.1111/andr.13439, PMID: 37038051

[32] Marcotorchino J Romier B Gouranton E Riollet C Gleize B Malezet-Desmoulins C. Lycopene attenuates LPS-induced TNF-α secretion in macrophages and inflammatory markers in adipocytes exposed to macrophage-conditioned media. Mol Nutr Food Res. (2012) 56:725–32. doi: 10.1002/mnfr.201100623, PMID: 22648619

[33] Doseděl M Jirkovský E Macáková K Krčmová LK Javorská L Pourová J. Vitamin C-sources, physiological role, kinetics, deficiency, use, toxicity, and determination. Nutrients. (2021) 13:615. doi: 10.3390/nu13020615, PMID: 33668681 PMC7918462

[34] Jiang Q. Natural forms of vitamin E: metabolism, antioxidant, and anti-inflammatory activities and their role in disease prevention and therapy. Free Radic Biol Med. (2014) 72:76–90. doi: 10.1016/j.freeradbiomed.2014.03.035, PMID: 24704972 PMC4120831

[35] Eleazu C Obianuju N Eleazu K Kalu W. The role of dietary polyphenols in the management of erectile dysfunction-mechanisms of action. Biomed Pharmacother. (2017) 88:644–52. doi: 10.1016/j.biopha.2017.01.125, PMID: 28142121

[36] Liu RJ Li SY Xu ZP Yu JJ Mao WP Sun C. Dietary metal intake and the prevalence of erectile dysfunction in US men: Results from National Health and nutrition examination survey 2001–2004. Front Nutr. (2022) 9:974443. doi: 10.3389/fnut.2022.974443, PMID: 36407550 PMC9668876

[37] Hao X Chen X Ren C Pan Y Xu Z Wang Q. Association between composite dietary antioxidant index and erectile dysfunction: a cross-sectional study from NHANES. BMC Public Health. (2024) 24:3362. doi: 10.1186/s12889-024-20880-4, PMID: 39623347 PMC11613466

[38] Zhu H Chen S Ye Q Lin W Li T Xu Z. Association between composite dietary antioxidant index and erectile dysfunction among American adults: a cross-sectional study. Sci Rep. (2024) 14:21230. doi: 10.1038/s41598-024-72157-w, PMID: 39261605 PMC11390727

[39] Chen M Zhang Z Zhou R Li B Jiang J Shi B. The relationship between oxidative balance score and erectile dysfunction in the U.S. male adult population. Sci Rep. (2024) 14:10746. doi: 10.1038/s41598-024-61287-w, PMID: 38730004 PMC11087471

[40] Ruan Z Xie X Yu H Liu R Jing W Lu T. Association between dietary inflammation and erectile dysfunction among US adults: a cross-sectional analysis of the National Health and nutrition examination survey 2001-2004. Front Nutr. (2022) 9:930272. doi: 10.3389/fnut.2022.930272, PMID: 36438746 PMC9691656

[41] Zheng Y Liu W Zhu X Xu M Lin B Bai Y. Associations of dietary inflammation index and composite dietary antioxidant index with preserved ratio impaired spirometry in US adults and the mediating roles of triglyceride-glucose index: NHANES 2007-2012. Redox Biol. (2024) 76:103334. doi: 10.1016/j.redox.2024.103334, PMID: 39217849 PMC11402638

[42] Yang C-C Liao P-H Cheng Y-H Chien C-Y Cheng K-H Chien C-T. Diabetes associated with hypertension exacerbated oxidative stress-mediated inflammation, apoptosis and autophagy leading to erectile dysfunction in rats. J Chin Med Assoc. (2022) 85:346–57. doi: 10.1097/JCMA.0000000000000691, PMID: 35019864 PMC12755464

[43] Simental-Mendía LE Morales-Gurrola FG Barragán-Zúñiga LJ. The triglyceride-glucose index as a surrogate measure to assess glycemic control in type 2 diabetes patients. Ir J Med Sci. (2025) 194:515–520. doi: 10.1007/s11845-025-03893-9, PMID: 39869145

[44] Ramdas Nayak VK Satheesh P Shenoy MT Kalra S. Triglyceride glucose (TyG) index: a surrogate biomarker of insulin resistance. J Pak Med Assoc. (2022) 72:986–8. doi: 10.47391/JPMA.22-63, PMID: 35713073

[45] Contreras C Sánchez A Martínez P Climent B Benedito S García-Sacristán A. Impaired endothelin calcium signaling coupled to endothelin type B receptors in penile arteries from insulin-resistant obese Zucker rats. J Sex Med. (2013) 10:2141–53. doi: 10.1111/jsm.12234, PMID: 23875673

[46] Yilmaz M Karaaslan M Tonyali S Celik M Toprak T Odabas O. Triglyceride-glucose index (TyG) is associated with erectile dysfunction: a cross-sectional study. Andrology. (2021) 9:238–44. doi: 10.1111/andr.12904, PMID: 32936988

[47] Cersosimo E DeFronzo RA. Insulin resistance and endothelial dysfunction: the road map to cardiovascular diseases. Diabetes Metab Res Rev. (2006) 22:423–36. doi: 10.1002/dmrr.634, PMID: 16506274

