# Supplementary material for: Associations of dietary inflammatory index and composite dietary antioxidant index with erectile dysfunction and the mediating role of metabolic dysregulation: a cross-sectional analysis of NHANES 2001–2004 data
Source: Front Nutr. 2025 Jun 3;12:1538874. doi: 10.3389/fnut.2025.1538874 (PMC12170290; doi:10.3389/fnut.2025.1538874)
Supplement: Supplementary file 1 [file Table_1.DOCX]

**Supplemental File**

**eFigure 1.** Spearman's rank correlation coefficients between dietary quality indices and metabolic indices.

**eFigure 2.** Sankey diagram illustrated the links of the dietary quality index-metabolic indicators-ED.

**eTable 1.** Associations of dietary quality index with ED and metabolic indicators (after redefining the ED population).

**eTable 2.** Associations of metabolic indicators with ED (after redefining the ED population).

**eFigure 1. Spearman's rank correlation coefficients between dietary quality indices and metabolic indices.**

**
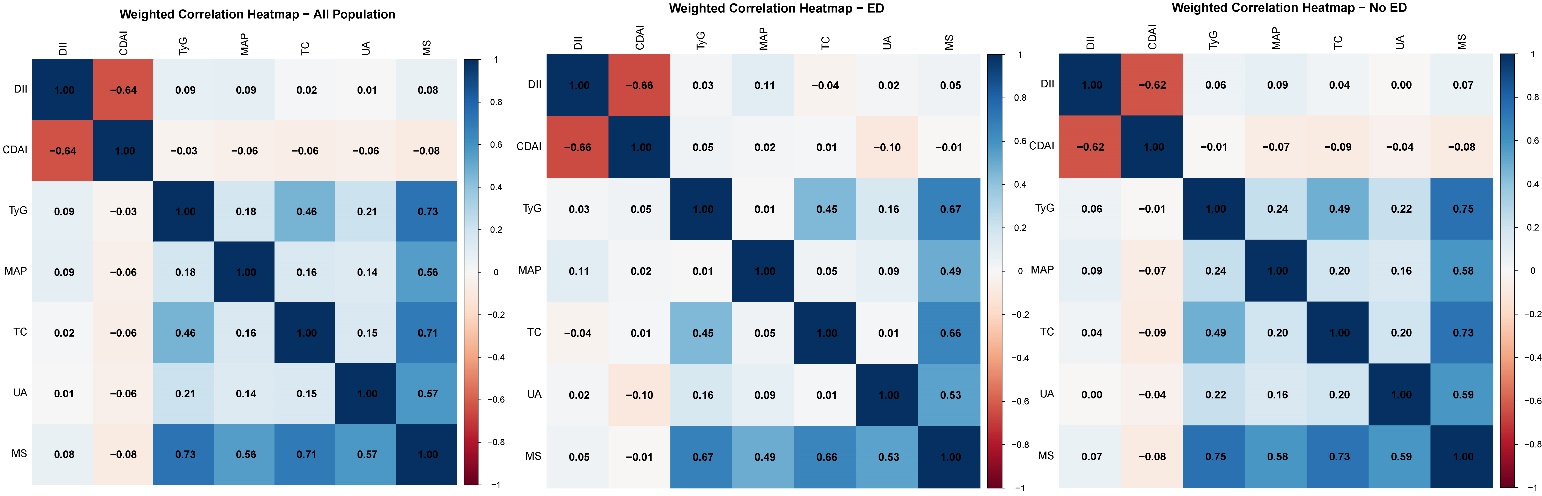
**

**eFigure 2. Sankey diagram illustrated the links of the dietary quality index-metabolic indicators-ED.**


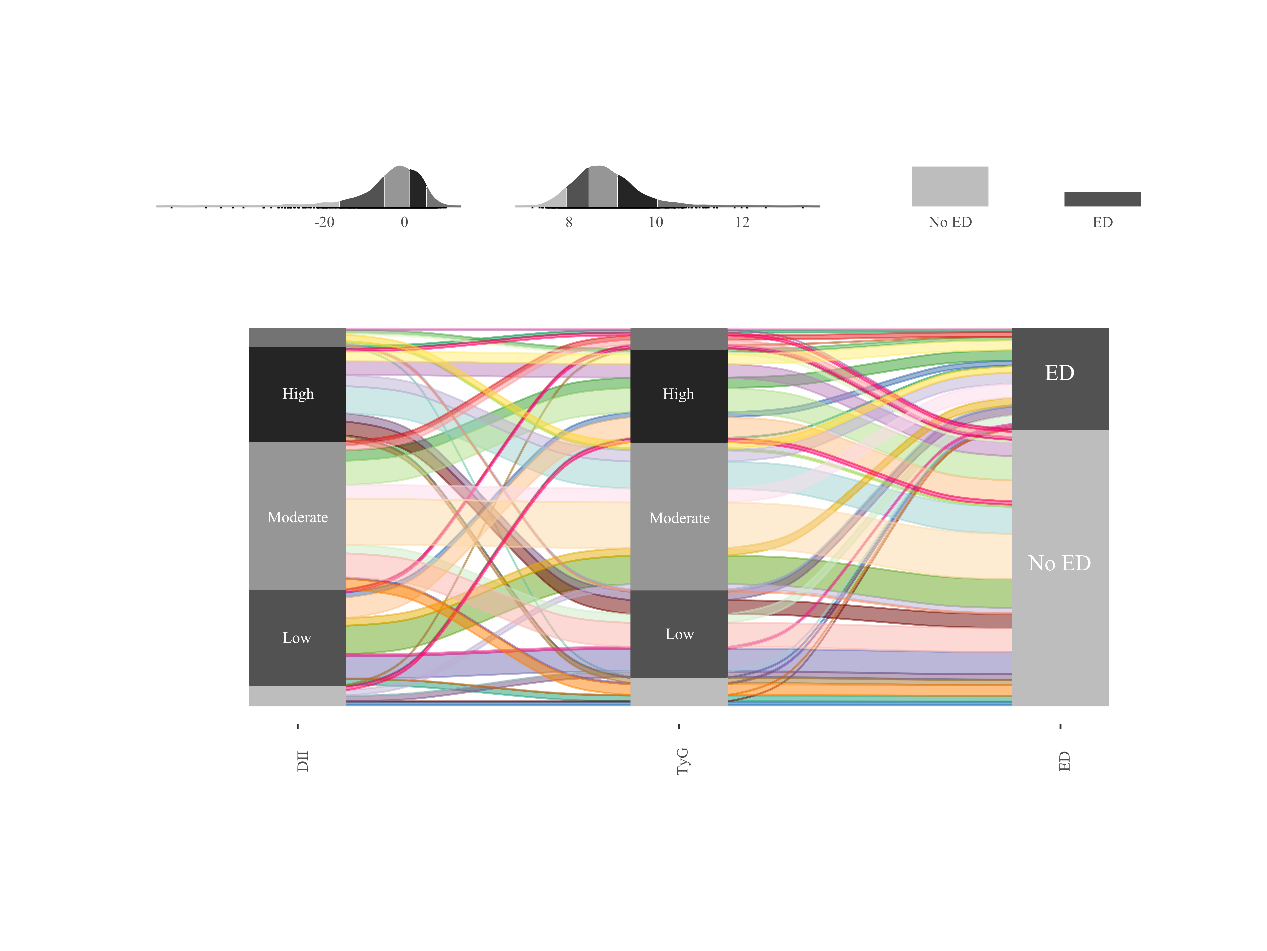


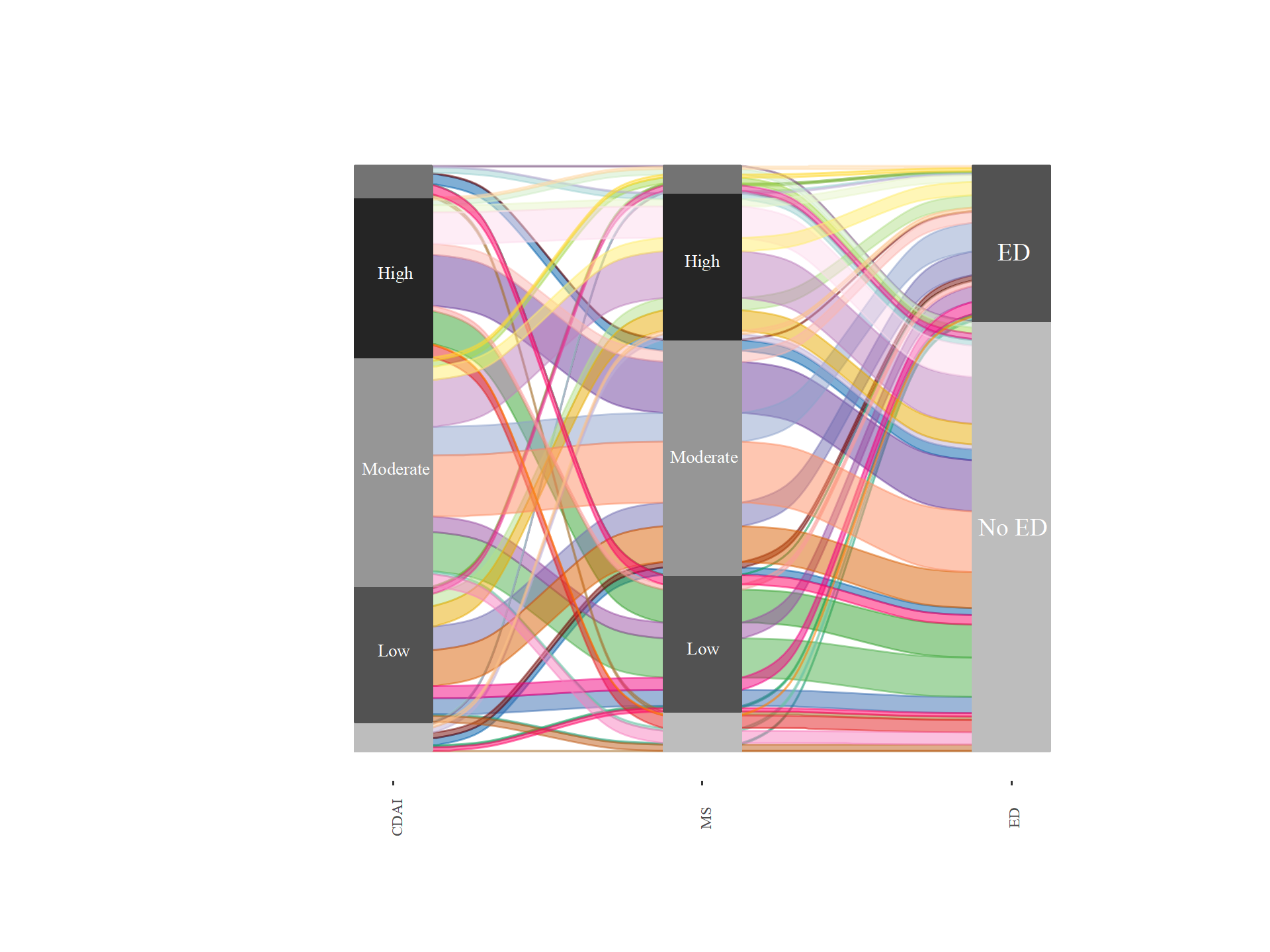


**eTable 1.** Associations of dietary quality index with ED and metabolic indicators (after redefining the ED population).

**Note:** Adjusted for ethnic, age, PIR (poverty-to-income ratio), BMI (body mass index), smoking status, physical activity, education level, and marital status.

| **Variables** | **β or OR (95CIs)** | ***P*** |
| --- | --- | --- |
| **ED** |  |  |
| DII | 1.08 (1.02,1.15) | **0.007** |
| CDAI | 0.95 (0.93,0.98) | **0.003** |
| **MAP** |  |  |
| DII | 0.08 (-0.01, 0.17) | 0.087 |
| CDAI | -0.03 (-0.10, 0.04) | 0.353 |
| **Uric acid** |  |  |
| DII | 0.001 (-0.01, 0.01) | 0.785 |
| CDAI | -0.01 (-0.02, 0.001) | **0.041** |
| **Cholesterol** |  |  |
| DII | 0.06 (-0.28, 0.41) | 0.711 |
| CDAI | -0.21 (-0.52, 0.10) | 0.168 |
| **TyG index** |  |  |
| DII | 0.01 (-0.003, 0.01) | 0.227 |
| CDAI | 0.001 (-0.004, 0.01) | 0.789 |
| **MS** |  |  |
| DII | 0.02 (-0.01, 0.04) | 0.160 |
| CDAI | -0.01 (-0.03, 0.004) | 0.132 |

**eTable 2.** Associations of metabolic indicators with ED (after redefining the ED population).

| **Metabolic indicators** | **β** | **SE** | **P**  **value** | **Odds**  **ratio** | **CI**  **(2.5%)** | **CI**  **(97.5)** |
| --- | --- | --- | --- | --- | --- | --- |
| MAP | -0.01 | 0.01 | 0.263 | 0.99 | 0.97 | 1.01 |
| Uric acid | 0.10 | 0.09 | 0.255 | 1.11 | 0.93 | 1.32 |
| Cholesterol | -0.003 | 0.003 | 0.355 | 1.00 | 0.99 | 1.00 |
| TyG index | 0.43 | 0.12 | 0.003 | 1.54 | 1.19 | 1.98 |
| MS | 0.03 | 0.04 | 0.454 | 1.03 | 0.95 | 1.11 |

**Note:** Adjusted for ethnic, age, PIR (poverty-to-income ratio), BMI (body mass index), smoking status, physical activity, education level, and marital status.
